# The 2005 census and mapping of slums in Bangladesh: design, select results and application

**DOI:** 10.1186/1476-072X-8-32

**Published:** 2009-06-08

**Authors:** Gustavo Angeles, Peter Lance, Janine Barden-O'Fallon, Nazrul Islam, AQM Mahbub, Nurul Islam Nazem

**Affiliations:** 1 MEASURE Evaluation Project, University of North Carolina at Chapel Hill, USA; 2 Department of Maternal and Child Health, University of North Carolina at Chapel Hill, USA; 3 Centre for Urban Studies, Dhada, Bangladesh

## Abstract

**Background:**

The concentration of poverty and adverse environmental circumstances within slums, particularly those in the cities of developing countries, are an increasingly important concern for both public health policy initiatives and related programs in other sectors. However, there is a dearth of information on the population-level implications of slum life for human health. This manuscript describes the 2005 Census and Mapping of Slums (CMS), which used geographic information systems (GIS) tools and digital satellite imagery combined with more traditional fieldwork methodologies, to obtain detailed, up-to-date and new information about slum life in all slums of six major cities in Bangladesh (including Dhaka).

**Results:**

The CMS found that Bangladeshi slums are very diverse: there are wide intra- and inter-city variations in population size, density, the percent of urban populations living in slums, and sanitation conditions. Findings also show that common beliefs about slums may be outdated; *of note*, tenure insecurity was found to be an issue in only a small minority of slums.

**Conclusion:**

The methodology used in the 2005 Bangladesh CMS provides a useful approach to mapping slums that could be applied to urban areas in other low income societies. This methodology may become an increasingly important analytic tool to inform policy, as cities in developing countries are forecasted to continue increasing their share of total global population in the coming years, with slum populations more than doubling in size during the same period.

## Background

For the first time in history, the majority of humanity lives in urban areas. Currently, the world's urban population accounts for more than 3 billion people; over the next 25 years, the figure is projected to increase to nearly 5 billion [1]. Much of this growth will occur in "less developed" nations (as defined by the United Nations Population Division (UNDP)), particularly in Asia and Africa, where urban populations will more than double [1]. Slum populations are expected to grow roughly in line with the overall global urban population, increasing from 924 million to 2 billion over the next three decades [2]. In recognition of their increasing importance as urban environments, slums were explicitly addressed in the Millennium Development Goals (MDG) through target 11, which aims to improve the lives of a minimum of 100 million slum dwellers by 2020.

Bangladesh will be no exception to these urban growth trends. The rapid growth of urban areas is already apparent in Bangladesh, a country historically characterized by densely populated rural areas. Bangladesh has a 2005 population of 153 million; 39 million of whom, or 25%, are living in urban areas. The urban population of Bangladesh is projected to nearly double over the next 20 years; in contrast, the rural population will grow by less than 15 percent [1]. Dhaka, with an estimated 2005 population of 9.1 million, is one of the largest and fastest growing cities of the world. The greater Dhaka urban agglomeration is projected to grow at an annual average rate of 2.72 during the period 2007–2025, making it the fastest growing "mega-city" in the world [3].

Unsurprisingly, slum life and its consequences for health have attracted empirical research attention. This has included both attempts to estimate broad health profiles for slum populations and to study some particular causal or distributional dimension of slum life. Some of this work has relied on samples representative of slum populations at some broad (eg city-wide or national) level. For instance, a cross-sectional, DHS-type survey of Nairobi slums generated a health profile that permitted comparisons of indicators between slums and other domains in Kenya [4]. There have also been studies (eg Daniere and Takahashi's investigation of poverty and access in slum communities of Bangkok [5]) that have focused on particular channels of human welfare using samples based on some variety of slum sample frame (Daniere and Takahashi rely on a pre-existing slum list from Setchell (1992) [6]).

Broadly representative slum samples have, however, generally been a scarce resource. The underlying reason for the dearth of representative samples for slum populations has been the paucity of sample frames capable of supporting explicit slum domains. There are a variety of conceivable responses to this challenge available to the empirical researcher. One is to construct a sample frame as a first step to the study. However, given the expense of this, it is likely often to be feasible only within selected****neighborhoods or other subdivisions of cities. For instance, Pryer (2003) constructed a sample frame of 117 slum communities in Mohammadpur thana of Dhaka City, from which 25 primary sampling units of 20–50 households each were selected for inclusion in a sample of roughly 850 households [7]. Another approach has been to rely on case studies using one or more slums [8-13]. An obvious potential drawback to either building a sample frame for small areas of cities or pursuing the case study approach is that the broader representativeness of results based on such narrow slices of slum society is unclear.

Another approach is to adapt existing nationally representative household samples to slum analysis. An obvious resource for such an approach are census or DHS-type samples (and hence census- or DHS-type clusters, which are unfortunately generally not based on any prior attempt at assignment to slum and non-slum domains), with which one could strive to identify slum clusters or households with the information available from existing survey instruments. Examples include innovative applications of this information to fashion household-level slum indices using principal components-related methodologies [14].

The drawback to such adaptations of existing samples that are representative at the national (or, for instance, the urban or city) level is that the primary sampling units/clusters underpinning these samples were not designed for stratification along slum/non-slum lines. In many societies such as Bangladesh (where slums are often small and compact but nonetheless frequently exhibit an irregular, sprawling layout) these clusters likely straddle slum and non-slum areas. Thus it becomes problematic to identify a cluster as a slum, even if a simple majority of households within it are identified as slum households. Moreover, from a conceptual standpoint, slum definitions based on household-level indices are somewhat limiting (slum is clearly a community-level concept).

Thus, owing to the scarcity of slum sample frames at city or national levels, many empirical examinations of slum life are compelled either to confine attention to small areas of cities or to somehow adapt existing household samples not designed for stratification on slum lines. Those that have been able to rely on broad sample frames [4] are comparatively rare. Unfortunately, the level of information regarding the broad (ie city- or country-wide) health profiles of slum populations does not even approach that available for national populations as a whole through household surveys such as the DHS.

All of these considerations have historically applied in Bangladesh, where slums are an increasingly important focal point for policy initiatives and programs. Motivated by such considerations, the Government of Bangladesh and the United States Agency for International Development (USAID) desired a study of health in urban areas of the country that included a representative sample of urban slums. As a first step to achieving this goal, we conducted the 2005 Census and Mapping of Slums (CMS) in the six main cities of the country. The CMS used a number of steps to produce accurate and detailed maps of slum and non-slum areas that were combined with survey information to produce a database with information on the location, estimated population sizes, and essential characteristics of all urban slums in Bangladesh. The CMS provided the sample frame for the Urban Health Study (UHS). However, the findings from the CMS are by themselves valuable and in some instances challenge common assumptions about slum characteristics. In this manuscript we describe the procedures used for the 2005 CMS, the key findings, and some of the applications to programs operating on the ground that have occurred since its completion.

## Methods

The 2005 Census and Mapping of Slums (CMS) was conducted by a team of researchers from the Centre for Urban Studies (CUS), Associates for Community and Population Research, the National Institute of Population Research and Training, USAID, the International Centre for Diarrhoeal Disease Research, Bangladesh (ICDDR, B), and the University of North Carolina at Chapel Hill. The purpose of the CMS was to produce a nationally representative sample frame for the six City Corporations of Bangladesh (the major cities that serve as the seats for the six administrative divisions of the country). The design of the CMS was guided by two considerations, the first of which was to obtain primary sampling units that revolved around a behaviorally meaningful notion of urban community. The CMS was thus designed to deliver a slum sample frame characterized by slum clusters that reflected, to the greatest extent possible, coherent neighborhoods. Second, the 2005 CMS was intended to serve as a tool that could be leveraged by planners and program officers, who could use it to aid the targeting of urban health and other human welfare-related programs to concentrated areas of poverty and poor environmental circumstances. The 2005 CMS was conducted in three phases.

### Phase 1: Base Map Preparation Using Satellite Image

The first step of phase 1 involved the development of baseline maps of the City Corporations to identify suspected slum settlements and provide an accurate overall organizing framework. CUS collected publicly-available maps from the Survey of Bangladesh (SOB), a government organization in the Ministry of Defense that surveys and compiles maps. The baseline maps were then digitized using GIS (Arcinfo, Arc GIS) software.

The next step involved procuring commercially available IKONOS 2003 satellite images for Dhaka (panchromatic images with 1-meter resolution) and IRS 2001–2003 images for Chittagong, Khulna, Rajshahi, Sylhet and Barisal City Corporations (5-meter resolution). These maps were the most recent, highest resolution images available. The satellite images were then geo-referenced (meaning that clearly identifiable points and landmarks on the images were associated with precise GPS coordinates gathered during initial field visits). Coordinates were initially collected in WGS84 then converted to BTM (Bangladesh Transverse Mercator) because it became apparent that the close proximity of some slum clusters in the dense urban environment of Bangladesh caused some slum polygons to overlap. BTM is a standard in Bangladesh. Gathering a sufficient number of reference points (typically 4 to 8 per ward) allowed the images to be characterized by precise latitudinal/longitudinal positions (root mean square error in geo-referencing was .005).

The images were then used to update the original SOB maps, which contained streets, waterways and local landmarks but were often inaccurate because they did not capture more recent development, which can be substantial in highly dynamic cities such as Dhaka. Less frequently, but surprisingly, the SOB maps occasionally failed to capture accurately even established areas. As a result, the process of updating the streets, boundaries and other features on the SOB maps by reconciling them with the satellite images produced the only fully accurate street maps of the six cities (occasionally, small revisions were made to the streets or other boundaries on the maps after field teams discovered discrepancies).

The satellite images were also used to identify suspected slum settlements. This visual assessment focused on settlement density and building materials. The two key characteristics were density and roofing materials (the two attributes most obvious from above). However, the specific standards varied from city to city and even within areas of some cities because of the variation in relative density patterns and typical roofing materials in slums. Suspected slums were located and delineated on the corrected SOB maps, which then became the basis for the second phase. The final base maps contained roads, waterways, boundaries, suspected slum settlements and, when useful, referenced local landmarks.

The satellite images were not intended to identify all slum settlements accurately, but instead to provide an organizing framework for Phase 2 (fieldwork for "ground truthing"). The investigators involved in earlier slum censuses (such as a 1996 census and mapping of slums in the Dhaka City Corporation and Metropolitan Authority) experienced an extremely chaotic, costly and prolonged first stage of fieldwork, in which field teams struggled with inaccurate maps of their operational areas and no prior sense of where potential slum concentrations might be. The satellite images provided crucial accurate prior information regarding the street layout of the city corporations and the location of likely slum concentrations. The age of the satellite images was not an issue for generating accurate street maps: what remained for field teams, if anything, were far smaller, rarer discrepancies between their guide maps and the physical circumstances of their operational areas which were easily rectified without confusion.

A fully accurate picture of the location of possible slum concentrations could not be attained with satellite or aerial photos alone, as there was no formal rule or signature that could be applied to recover exact pictures of slum conditions on the ground. While the majority of slums did conform to the density and roof patterns of slums in their locale, a substantial minority (30 percent) were missed. Occasionally this was due to the age of the images, but more often to the fact that there was diversity to slum circumstances: for example, while there tended to be a dominant roof type in the slums of each city, a household survey in slum and non-slum areas that followed this study found a non-trivial number of exceptions to this dominant pattern. The density and roofing material criteria most relevant to slum identification from above could also readily lead to mis-identification (as markets, meat processing plants, light industrial facilities, etc. are mistaken for slums). These challenges could only be overcome through ground truthing. For these reasons it was decided not to develop and document a formal signature by which the slums identified through our satellite image analysis could be recovered by others: it would only yield a partial and not wholly accurate picture of slums in the study cities. The validity of the slum identification rested ultimately on the ground truthing.

In principal, more recent and accurate images, such as QuickBird images from DigitalGlobe (.6 m panchromatic), could have been used, but these would have added to cost for little benefit. They could not satisfactorily prevent the mis-identification problem, which could be overcome only through street-level inspection; a perspective that even the highest resolution images cannot provide. In many of the city corporations (or portions thereof) included in the study, heavy tree cover limits the value of still higher resolution pictures. However, these are all factors particular to the circumstances of urban Bangladesh. It could be that in other low-income societies, ground conditions and slum environmental circumstances might render higher resolution images more valuable.

### Phase 2: "Ground Truthing"

In the second phase, referred to as "ground truthing", teams went into each ward of the six cities, including the larger Dhaka Metropolitan Authority, to assess ground conditions. Ground truthing was necessary for several reasons. First, it was used to confirm suspected slum settlements identified in Phase 1. For instance, poultry processing facilities often looked like slums from the satellites. Second, the teams checked for slum settlements not obvious from the images. In areas that were generally flat and exhibited little tree cover, the satellite images revealed the overwhelming majority of slums. In settings where the landscape was characterized by steep gradients or heavy foliage, more slum communities were missed in Phase 1. Finally, although the most recent satellite images had been used in Phase 1, some slums had disappeared while others had formed since the images were taken. If an urban area was comprised of at least 10 households and appeared to be a slum from satellite images, field investigators entered it to complete a checklist. In the case of mess concentrations (basically dormitory-type environments) this threshold was developed a bit further. A mess 'household' was defined as a group within a mess environment that shared a kitchen. Occasionally, there was more than one household, so defined, in a single mess structure/building (though we do not believe that there was a single instance where a single continuous, self-contained mess structure was defined as a slum). Field teams also found instances where such mess 'households' were actually spread among more than one mess structure. Far more commonly, there was one household, so defined, per mess structure.

Mess structures are often tightly concentrated in a small area. When that concentration of mess structures held more than 10 households (defined as described) and met the scientific criteria for slums in this study (to be outlined shortly), it was declared a slum. Isolated mess structures (which rarely had more than two households, per the definition of the household applied to the mess environment) were not treated as slums.

The checklist was used by the field workers to provide a comprehensive description of the general conditions in suspected slum settlements, including estimates of population size. It also provided field teams with an opportunity to confirm the boundaries of the settlement. The checklists were administered to at least three informants in each suspected slum community.

Informants were individuals with some education and at least five years tenure in the slum (or had lived in the slum from its founding) who could be regarded as central figures (community leaders, teachers, shop keepers, NGO workers, etc.), whose specific background depended on the nature of the slum. In the larger slums (which tended to require far more time consuming ground truthing), fieldworkers divided the slum into segments and interviewed different informants in each segment to make sure that the overall picture from the slum conformed with these 'local' perspectives. Nonetheless, there is the possibility for some bias by relying on the opinions of community-level informants.

On completion of the checklist, field team members declared a community to be a slum if it met four of five basic conditions: 1) Poor housing conditions (defined using guidelines from the literature); 2) High overall population density (using the widely suggested threshold of 300 persons per acre and predominantly (over 75% single room occupancy); 3) Very poor sanitation and inadequate water access; 4) High prevalence (over 75%) of people with income below the poverty level, or a monthly income <5,000 Taka, and 5) insecure land tenure.

It is important to recognize that this approach pursued a consistent definition for slums across city corporations. However, in some respect the concept of slum may vary across or even within cities. Thus, our approach in some sense imposes a uniformity to the notion of a slum that may not always be appropriate in Bangladesh. Nonetheless, it is also important to recognize that the alternative course (more 'local' slum definitions) is not without problems either. To begin with, one would need a considerable amount of prior information about slums, more than exists for many cities or sections in Bangladesh. Moreover, it would have to be timely: even in areas where there is a considerable body of research, slum circumstances are constantly evolving. Our slum definition reflects the minimum common denominator for characteristics associated with slums throughout Bangladesh. We thus decided that is was more practical to pursue a minimum relevant standard for purposes of scientific consistency.

The satellite images, ground truthing, and other technologies (e.g. GPS receivers) available as research tools since the last census and mapping of slums (in Dhaka in 1996) facilitated rich linkages between data sources, and contributed to far fewer and more easily detectable errors. By exposing errors in the SOB maps before fieldwork, for instance, the satellite images allowed for the updating and preparation of reliable baseline maps in a controlled, deliberate and centralized fashion. This effectively eliminated one channel of feedback from the field (i.e. field workers reports of difficulties with the SOB maps) that had in past efforts proved distracting and confusing.

### Phase 3: Data Processing

The information from the checklists was entered into a geographical database at CUS headquarters. This process involved a number of detailed and redundant consistency checks. Problematic checklists were identified and re-examined. When necessary, field teams re-visited the settlement. From this process, a list of slums and their attributes was produced for each of the six City Corporations. Finally, our maps were again updated to reflect the final determination of the location and boundaries of slum settlements.

#### Timeline and Study Team

Work on the CMS proceeded on a city-by-city basis: in each city, personnel at the Centre for Urban Studies completed phase 1 of the study (construction of accurate maps and identification of possible slums) and then proceeded immediately to the fieldwork in that city. This had the advantage that staff at the central office was focused on the specifics of the city in which fieldwork was progressing. Toward the end of fieldwork in one city, map preparation and slum identification from satellite photos (ie phase 1) began for the next city. Typically, the ratio of time spent in fieldwork to time spent in base map preparation was about three or four to one, but there were exceptions (the two phases took about as long in Barisal at three weeks each).

In terms of personnel, the two senior investigators at the Centre for Urban Studies (CUS) have been involved in virtually every slum census and mapping done in Bangladesh, as well as a wide array of other applied urban geography studies. They oversaw all aspects of the study, splitting their time between the central office in Dhaka and the field. In the central office in Dhaka, two senior managers with long experience in GIS work (including slum studies) at CUS oversaw the work of five individuals, each with at least five years of experience at CUS in GIS and aerial analysis work, as they built base maps before fieldwork and then formed final slum maps and lists as fieldwork in each ward (and union, in the case of the Dhaka Metropolitan Authority) was completed. Finally, in the field five supervisors with a minimum of five years experience oversaw 30 investigators (each with a formal background in geography) working in 15 teams of 2. This small number of teams and heavy degree of supervision (by field supervisors as well as the senior investigators at CUS) provided for far tighter quality control. Finally, the tools of the field personnel included the base maps, GPS devices and other essential materials.

## Results

### Outputs of Census & Mapping of Slums

**Figure 1:**
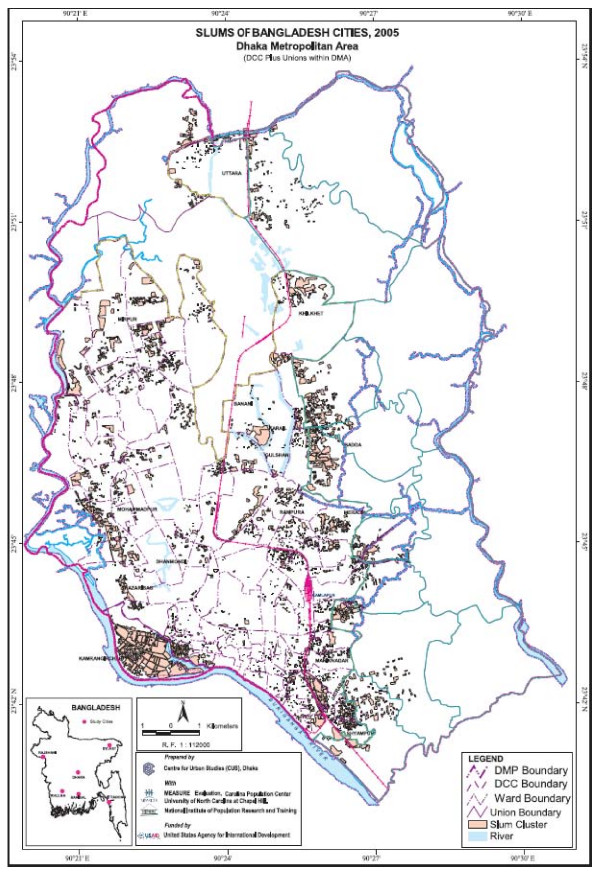
**Slum map of Dhaka**.

**Figure 2:**
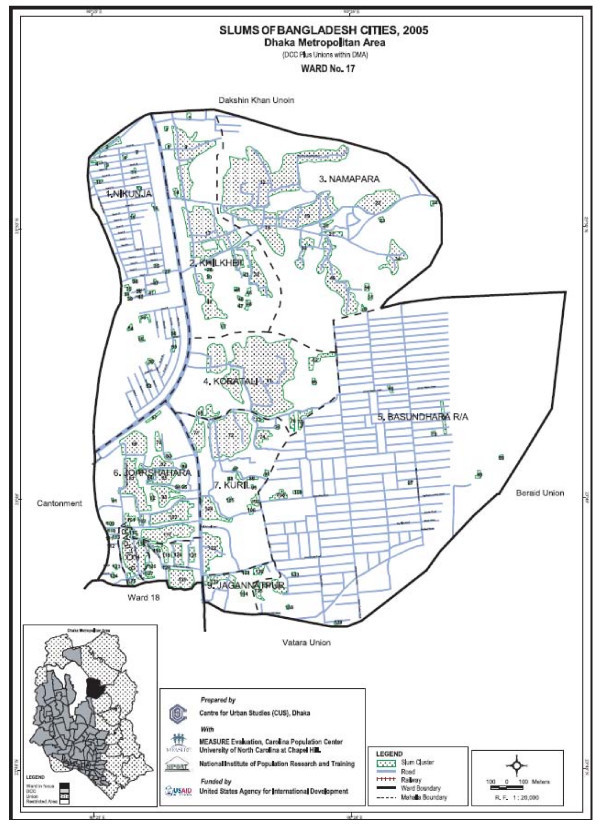
**Ward-level (Dhaka ward 17) slum map**.

This project produced a number of very valuable outputs, including:

1. Highly accurate, detailed ward and sub-ward (mohalla) level maps of slum settlements in the six City Corporations of Bangladesh. Examples of these are provided in Figure 1 and Figure 2.

2. A database describing the exact location of the settlements visited by field teams, as well as their general characteristics. This provides more detail than a simple slum/non-slum distinction. The information in the database is integrated with that in the maps, allowing, for instance, maps based on alternative slum designations to be generated quickly and easily.

3. More accurate street maps of the six City Corporations than were available.

4. A detailed report summarizing findings of the survey [15].

5. A website (at ) through which these materials are made available to the general public. Note that the online maps and spreadsheets disaggregate the Dhaka slum lists into those found in the wards of the Dhaka City Corporation (DCC) and those located in the unions of the Dhaka Metropolitan Authority (DMA). The DCC is the administrative authority for the strictest definition of the city of Dhaka while the DMA covers the areas just outside of the DCC. The DMA was included in the census and mapping to capture slum conditions closer to the dynamic urban periphery of the Dhaka megacity as well as to achieve at least some limited degree of comparability with a 1996 census of the slums of Dhaka which had included the DMA.

The outputs thus provide comprehensive pictures of the location and information on the basic characteristics of all slums in the six City Corporations in a manner that is readily accessible to the public.

### Key Findings

The Census and Mapping of Slums (CMS) involved the collection of a comprehensive checklist of slum characteristics that captured their basic demographic, socioeconomic and environmental circumstances. Table 1 provides some basic statistics regarding overall slum populations and population densities. We identified 9,048 slum communities across the six cities. Unsurprisingly, by far the most (4,966) were in Dhaka. Dhaka also had the largest slum population. While the rank of the cities by total slum population mirrored that by their overall populations, the proportion of the city population living in slums ranged from 19.5% in Khulna to nearly 40% in Dhaka.

**Table 1:** The slums of the six city corporations: basic characteristics

**City**	**a**	**b**	**c**	**d**	**e**	**f**	**g**	**h**	**i**	**j**
Dhaka Metropolitan Area	4,966	9,136,182	3,420,521	**5,715,661**	**1.67**	37.4	29,857	220,246	**19,677**	**0.089**

Chittagong	1,814	4,133,014	1,465,028	**2,667,986**	**1.82**	35.4	23,299	255,100	**15,543**	**0.061**

Khulna	520	966,837	188,442	**778,395**	**4.13**	19.5	20,346	132,988	**16,884**	**0.127**

Rajshahi	641	489,514	156,793	**332,721**	**2.12**	32.0	9,544	67,236	**6,796**	**0.101**

Barisal	351	365,059	109,705	**255,354**	**2.33**	30.1	7,152	133,730	**5,084**	**0.038**

Sylhet	756	356,440	97,676	**258,764**	**2.65**	27.4	12,961	154,741	**9,630**	**0.062**

a) Number of slum communities

b) Overall 2005 population (estimate)

c) Slum population

d) Non-slum population

e) Ratio of non-slum to slum population

f) Slum population as a percent of city population

g) Overall population density (persons per Km^2^)

h) Slum population density (persons per Km^2^)

i) Non-slum population density (persons per Km^2^)

j) Ratio of non-slum to slum population density

Overall city-wide densities in Dhaka and Chittagong were very high (for reference, the population density of New York City is approximately 10,000 persons per km^2^). Nonetheless, density levels in the slum neighborhoods were far higher, though they varied considerably across the City Corporations. Despite the fact that most of the urban population lived outside of the slums, non-slum areas were far less densely settled. Although not illustrated by Table 1, slum population densities also exhibited pronounced within-city variation, as different slums in Dhaka, for instance, had vastly different densities.

**Figure 3:**
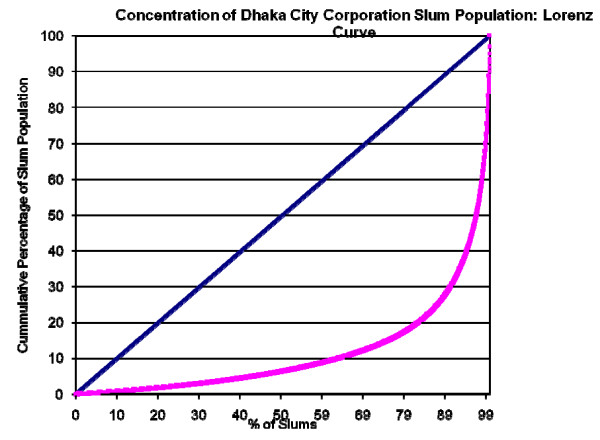
**Concentration of Dhaka City Corporation slum population: Lorenz curve**.

**Figure 4:**
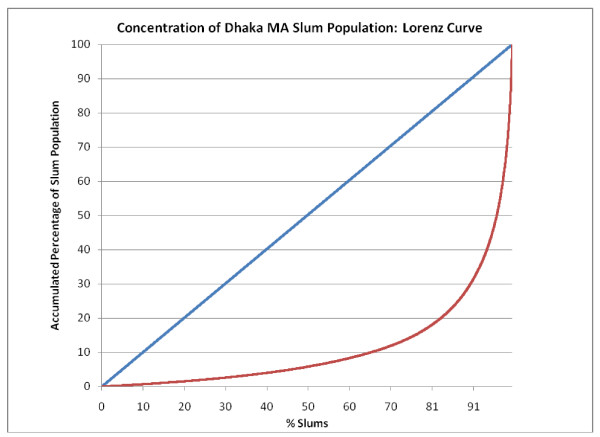
**Concentration of Dhaka Metropolitan Authority (MA) slum population: Lorenz curve**.

Table 2 presents the distribution of slum communities by population size. The majority had 200 or fewer persons. Nonetheless, a non-trivial proportion of slums were larger. While the distribution by population-size exhibited the same general patterns across the six cities, there were notable differences between them. One important example was the largest slums (by population size), which were generally a phenomenon of the larger cities. For example, see Figure 3, which presents a Lorenz curve for the Dhaka City Corporation slum population, which shows that a few, very large slums accounted for the majority of the Dhaka slum population: only 3.2% of slums (n = 140) accounted for 50% of the slum population, whereas almost two-thirds of the slums (63.5%, n = 2,756) held only 10% of the slum population. (Figure 4 provides a Lorenz curve for the entire Dhaka Metropolitan Authority.)

**Table 2:** Distribution of slums by population size

**Slum Population Size (persons)**	**Dhaka**	**Chittagong**	**Khulna**	**Rajshahi**	**Barisal**	**Sylhet**	**Total**
Up to 100	39.0	24.4	45.6	47.7	33.6	58.3	38.5

101–200	22.3	21.5	25.4	28.2	33.3	31.5	24.0

201–300	9.4	12.3	6.3	9.5	12.5	5.9	9.7

301–400	4.9	6.8	4.4	4.2	4.0	1.6	4.9

401–500	3.2	4.0	4.0	1.1	5.4	0.8	3.2

501–1,000	8.4	11.7	7.9	5.0	4.8	1.5	8.1

1,001–2,500	7.3	13.8	4.6	3.4	4.3	0.3	7.4

2,501–5,000	2.8	3.1	0.6	0.6	1.4	0.1	2.3

5,001–10,000	1.6	1.9	1.0	0.2	0.6	0.0	1.3

Above 10,000	1.0	0.4	0.2	0.0	0.0	0.0	0.6

Total (%)	100.0	100.0	100.0	100.0	100.0	100.0	100.0

Number of slum cluster	4,966	1,814	520	641	351	756	9,048

**Figure 5:**
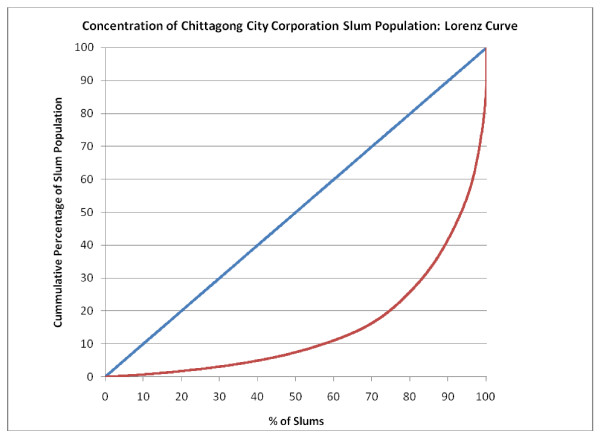
**Concentration of Chittagong City Corporation slum population: Lorenz curve**.

**Figure 6:**
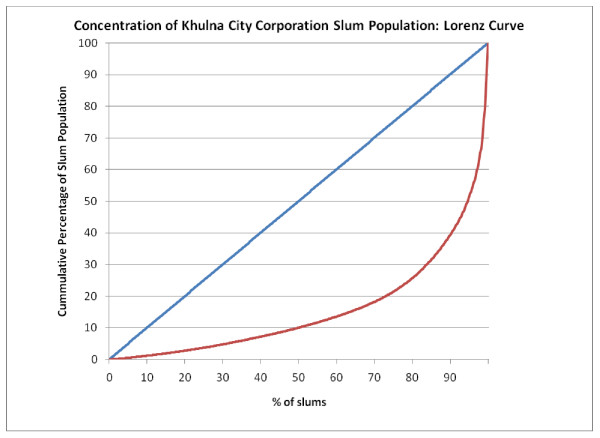
**Concentration of Khulna City Corporation slum population: Lorenz curve**.

**Figure 7:**
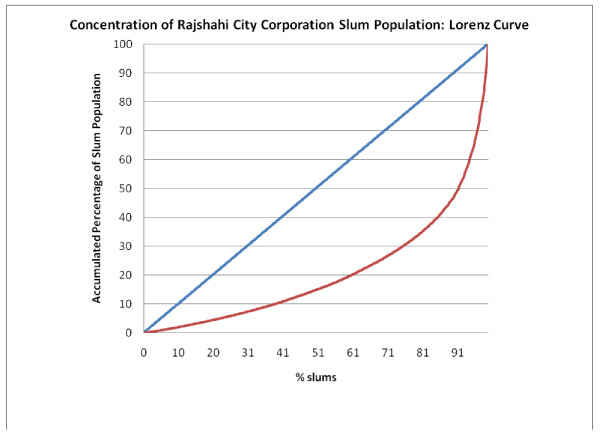
**Concentration of Rajshahi City Corporation slum population: Lorenz curve**.

**Figure 8:**
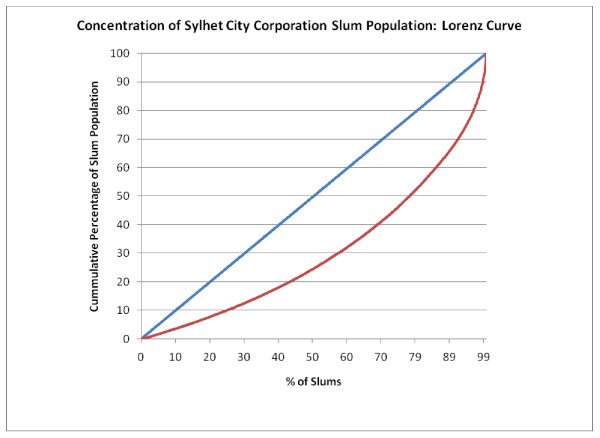
**Concentration of Sylhet City Corporation slum population: Lorenz curve**.

**Figure 9:**
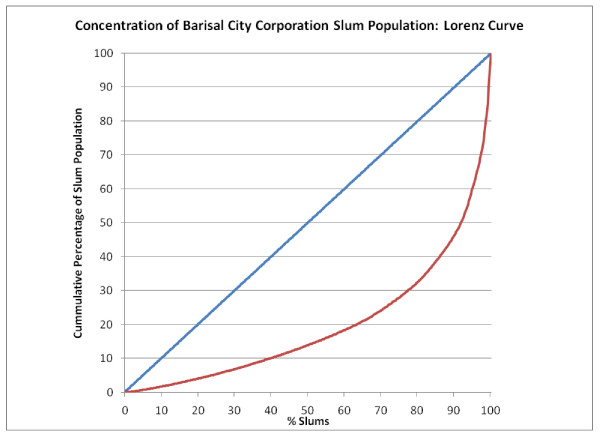
**Concentration of Barisal City Corporation slum population: Lorenz curve**.

Figure 5, Figure 6, Figure 7, Figure 8 and Figure 9 provide such Lorenz curves for the remaining city corporations. Table 3 provides the Gini coefficients associated with these various Lorenz curves. The Gini coefficients measure the level of inequality in the distribution of the slum population by slum size: higher values indicate a high concentration of the slum population in few slums, accompanied by large number of small slums. This was the case of Dhaka where, as one can see from its respective Lorenz curves, there was a high concentration of the slum population in few large slums together with a large number of small slums dispersed throughout the city. In Dhaka, 5.7% of the slum population (almost 200,000 people) lived in 2,483 slums (50% of all slums in the city). On the other hand, 50% of the slum population was concentrated in only 3.7% of slums (185 slums). The Gini coefficient seems to have increased with the size of the city. The lowest Gini was observed in Sylhet, which had the smallest population size, while the highest was in Dhaka.

**Table 3:** Gini coefficient by city

	Gini Coefficient	Total Population 2005 (Est.)	Slum Population 2005	Total Number of Slums
Dhaka	0.771	9,136,182	3,420,521	4,966

Chittagong	0.704	4,133,014	1,465,028	1,814

Khulna	0.687	966,837	188,442	520

Rajshahi	0.589	489,514	156,793	641

Sylhet	0.393	356,440	97,676	756

Barisal	0.607	365,059	109,705	351

**Figure 10:**
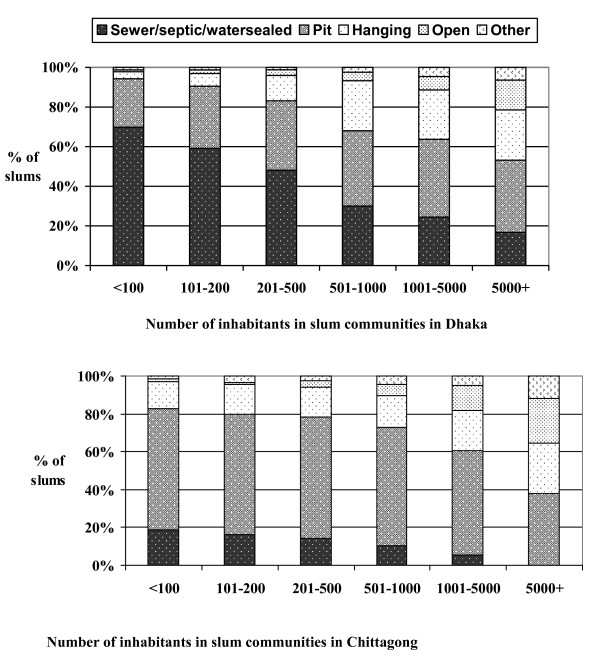
**Type of Latrine by Slum Population Size, Dhaka (N = 4,966) and Chittagong (N = 1,814)**.

These findings demonstrate that there was substantial inter and intra-city variation in the population sizes of slums. In this respect, population size and density were not exceptional: one of the general themes evident across the many indicators collected was the tremendous variation in slum circumstances across and within City Corporations. An example of inter- and intra-city variation in slum conditions is shown in Figure 10, which presents the type of latrine used by slum communities according to the population size of the community. In Dhaka, almost 70% of slums with 100 or fewer inhabitants had a latrine that was either linked to sewers or septic tanks or was water sealed. In contrast, less than 30% of slums with more than 500 inhabitants had these types of latrines, and only 17% of slums with populations of over 5,000 had sewer/septic or water sealed latrines. In Chittagong, pit latrines were much more common than in Dhaka and very few slum communities had sewer/septic or water sealed latrines (19% of slum communities of 100 or fewer inhabitants, 10% of slums with 501–1000 inhabitants and none of the slums of over 5,000).

A similar pattern of heterogeneity in slum conditions was observed across indicators. Over 60% of slum communities in Dhaka with fewer than 100 inhabitants had both a fixed place for garbage disposal and regular garbage collection. In contrast, less than 37% of slums over 500 exhibited these conditions, and only 25% of slums with more than 5,000 had a fixed place for garbage disposal while 20% have regular garbage collection. Chittagong showed generally worse conditions than in Dhaka: only about one-third of communities under 1,000 inhabitants had a fixed place for garbage disposal (compared to about one-quarter of slums with more than 1,000 inhabitants), and regular garbage disposal was provided for 27% of slums with under 1,000 inhabitants and 13% of slum communities with populations of over 1,000. While each indicator is interesting in its own right, this overall pattern is important because it reveals an essential truth of slum life: there is no such thing as a "typical" slum.

Traditional definitions of a slum (for research and policymaking purposes) in Bangladesh typically focused heavily on insecure property rights because so many slum communities had been illegally founded on public land. The 2005 CMS reveals the emerging centrality of privately owned slums with relatively secure tenure arrangements. For example, Table S1 shows that only 9 percent of slums in Dhaka were on public land while Rajshahi had the highest figure at 13.4 percent [Table S1, Additional file 1]. Only a small minority of slums in most communities reported tenure insecurity, assessed by whether the community has experienced an eviction or is currently under the threat of eviction. This suggests that much of the future growth in slum communities may occur on tracts where property rights are comparatively secure. One caveat is that in Dhaka at least, very large slums were vastly more likely to be on government land (31% for communities of over 5,000 compared to 4% of communities less than 100) and also more likely to experience tenure insecurity (18% vs. 2%).

Slums are also generally thought to be characterized by poor environmental circumstances and services. However, the picture emerging from this census is rather complex and suggests the need for more focused, as opposed to broad, policy responses. For instance, electricity and water from a municipal tap or tube well were actually widely available in the slums, but sanitation (gauged by garbage collection, toilet facilities and sharing of toilets) as per expectations, tended to be very poor [Table S1, Additional file 1]. Similarly, construction materials in slums were, unsurprisingly, poor; nonetheless, they tended to be much better in private slums, suggesting that as private tracts become the focus of slum life there might be some improvement in this regard with no policy intervention.

The CMS confirmed a generally held belief that slum communities are heavily populated with migrants from rural areas of the country. The CMS also provided new insights on the concentrated pattern of migration. For instance, only 6 of Bangladesh's 64 districts (Barisal, Comilla, Faridpur, Noakhali, Mymensingh and Chittagong) provided 54% of the total slum population across the six cities (see Table 4). Though Dhaka's slums attracted a significant number of migrants from 28 districts, nearly 53% of the Dhaka slum population came from only 5 of them: Barisal, Faridpur, Comilla, Mymensingh and Rangpur. Other cities experienced similarly concentrated migration. In some instances these tendencies can be explained (eg the districts are geographically close to the City Corporation), but on the whole, such focused urban migration in the face of widespread rural poverty suggests the need for more research into the subtle factors shaping rural to urban migration patterns in Bangladesh.

**Table 4:** Major districts of origin of the slum dwellers by city (percentage of slum dwellers in respective city)

**Dhaka (%)**	**Chittagong (%)**	**Khulna (%)**	**Rajshahi (%)**	**Sylhet (%)**	**Barisal (%)**	**All cities (%)**
Barisal, 22.7 Faridpur, 9.2 Comilla, 9.1 Mymensingh, 7.3 Rangpur, 4.6	Chittagong, 19.6 Comilla, 19.0 Noakhali, 14.9	Barisal, 35.9 Bagerhat, 17.9 Faridpur, 16.9	Rajshahi, 70.3	Mymensingh, 15.6 Sunamganj, 13.8 Comilla, 10.7 Rangpur, 9.5 Hobiganj, 9.5	Barisal, 65.3	Barisal, 19.4 Comilla, 11.0 Faridpur, 6.6 Noakhali, 6.2 Mymensingh, 5.5 Chittagong, 5.3

**Total 52.9**	**53.5**	**70.7**	**70.3**	**59.1**	**65.3**	**54.0**

Finally, although the 2005 CMS does not reveal trends in slum populations, some insights can be learned by comparing results for Dhaka with an earlier effort by the Centre for Urban Studies (CUS) [16]. Caution should be employed in considering such comparisons, as there were some conceptual and methodological differences in the censuses. For instance, there was far from complete overlap in the indicators included, and even in cases where they did overlap, the definitions were not always fully consistent. The latter complication is difficult to resolve since the 1996 data is not in readily accessible digital form nor do we have access. There were also differences in study protocols (the slum definition used in fieldwork in 2005 was a bit more elaborate, and it differed to a degree in its specifics). Finally, the definition of a slum evolves as urban conditions do, and while the period 1996–2005 may not seem long, it has witnessed substantial change in Bangladesh, as for instance standards for toilet facilities and drinking water sources have continued to shift. This suggests that the distinguishing characteristics of slums may also have done so. Nonetheless, some interesting points do emerge from a comparison of the 1996 and 2005 findings. These include:

1. A tremendous expansion occurred in the number of slums and populations living in them. In 1996, CUS found 3,007 slums in Dhaka with 1.1 million people (36% of the population at the time) living in them. Simple comparison of the 1996 and 2005 results thus suggest that the total population living in the slums of Dhaka essentially tripled, while the number of slum communities increased by roughly 70%.

2. Settlement density appears to actually fallen somewhat. In 1996, the average density for slums in Dhaka was 263,173 per square kilometer (1065 per acre). In 2005, the figure was 220,246 per square kilometer (891 per acre). It is possible that this is in part due to particularly intense slum formation in generally less densely settled peripheral zones, but this is difficult to establish with available data.

3. Housing quality in slums appears to have improved somewhat. For instance, in 1996 3.56 percent of slum housing structures in Dhaka were semi-pucca (essentially, walls made of brick or some similarly solid material but tin roofs). By 2005 that figure had risen tremendously to 52.3. It is our suspicion that this change was driven primarily by private slums.

4. The distribution of the ownership of slum land shifted heavily toward the private sector. In Dhaka in 1996, just over 77 percent of slum clusters and 48.8 percent of the slum households were on private land. By 2005 these figures were 89.8 and 70.3, respectively. This is one of probably one of the important trends behind our findings regarding tenure security in 2005.

5. Access to some essential services improved. For instance, in 1996 73.36 percent of slums had electricity while 30.36 had access to cooking gas. By 2005 these figures were 97.1 and 81.2 percent, respectively.

Thus, it is probably reasonable to conclude that between 1996 and 2005 there was a tremendous growth in the number of slums and size of slum populations (both of which were increasingly on private land) in Dhaka, but that slum population densities fell somewhat and slum housing structures and basic services improved. It is difficult to say, however, how large a role policies oriented toward the poor drove the changes in slum circumstances.

### Use of CMS for Planning and Program Purposes

Recognizing the heterogeneous circumstances of slums is of limited practical policy and programmatic use to planners and others if they cannot identify which slums exhibit various characteristics germane to their efforts. The Census and Mapping of Slums (CMS) generated detailed and accessible slum maps integrated with a database of slum characteristics, allowing for the quick identification and location of slums exhibiting certain combinations of circumstances. These tools have proven useful. A few major examples include:

1. The Bangladesh Rural Advancement Committee (BRAC) used the maps to place birthing huts in slums and to target expansion of their health program for the urban poor.

2. The Bangladesh AIDS program used the information to plan the future location of counseling and treatment centers for most at risk populations (eg sex workers, drug users, truck drivers, rickshaw pullers, etc.), who disproportionately reside in slums.

3. The United States Agency for International Development (USAID) funded Non-Governmental Organization (NGO) Service Delivery Program (administered by Pathfinder) used the maps to place main and satellite clinics for its next five year phase.

4. The United Nations Development Programme (UNDP) funded Local Partnership for Urban Poverty Alleviation requested the maps for use in targeting their efforts in their next 7 year phase.

5. The World Bank, which is currently working with the water and sewer authorities of the City Corporations to extend water and sanitation to the urban poor, requested the maps so that they can identify those slums not serviced by the present grid.

6. Family Health International (FHI)/Bangladesh intends to use the maps to track their own intervention sites and areas of program coverage for various programs.

In each instance, these programs were particularly interested in using the maps to identify slum clusters. The ready availability of information concerning the locations of slums can be an enormous asset in a setting like Bangladesh, where there are many slums and cataloging them for planning purposes would be a time consuming and expensive proposition for programs confronted with scarce resources. It provides programs with an accurate prior sense of the dimensionality of implementation in slums, and guides allocation decisions (eg facility placement to maximize slum residents or communities served). To our knowledge, the information on population characteristics was less commonly used.

## Discussion

The Bangladesh Census and Mapping of Slums (CMS) was designed to construct a sample frame for slum primary sampling units (PSUs) based on geographically coherent neighborhoods in the six main urban areas of Bangladesh. Specifically, it was intended to serve as a sample frame for an urban health survey in 2006, though in principal it could have been applied to any study treating slum and non-slum areas of the six city corporations as statistical domains. Nonetheless, as of this writing, it must be acknowledged that the 2005 CMS may now provide an increasingly dated representation of the slum environment in the six city corporations. Given the increasing importance of urban areas in general and slums in particular as a locus of poverty in societies such as Bangladesh, one could argue that this is an argument for more frequent slum mapping and census exercises to provide a current and relevant foundation for survey work in cities.

The CMS entailed the integration of advanced geographic tools (eg satellite imagery and GIS) with more traditional fieldwork-based techniques, a combination of methods that could be particularly useful in developing societies, where existing sample frame resources can be limited and there is often a substantive geographic component to survey domains of interest (due, for instance, to cluster-based sampling).

The CMS provided up-to-date and new information on slum life. Perhaps the most important finding is that there is no such thing as a "typical" slum in Bangladesh and, by extension, that conventional wisdom regarding slum life can be misleading. For instance, we found that tenure security was, as of 2005, no longer the urgent or central issue it had long been thought to be: the majority of slums were on privately owned tracts where the residents were typically renters with comparatively secure tenure rights. This finding supported anecdotal evidence garnered during the planning phase that public land had become subject to much stricter enforcement and that slums were increasingly founded on private land with much more secure tenure arrangements.

The principal indicators associated with MDG target 11 involve urban sanitation (the proportion of the urban population with access to improved sanitation) and security of tenure (the proportion of households with secure tenure). Our findings suggest that in this context, security of tenure is not a useful indicator of human welfare in Bangladesh in terms of its broad applicability (ie the proportion of slum dwellers with poor tenure security), nor is it obvious at this point that improvements in security of tenure in Bangladesh have meaningfully improved the welfare of slum dwellers.

The heterogeneity in slum conditions both within and across cities in Bangladesh has implications for targeting services. Whereas a large number of small slums can be more difficult to reach with public services, a few very large slums can create better conditions for targeting efforts. Our findings suggest that sanitation conditions in large slums, particularly the very large slums, tend to be worse in comparison to smaller slums.

The 2005 Bangladesh CMS is one among several efforts that has integrated modern GIS tools, digital imagery and more traditional fieldwork techniques in some combination or another in an attempt to more scientifically identify urban settlements. For example, a slum census in Pune, India assembled information into a GIS framework that linked digitized maps to a database of slum characteristics [17]. Hall et al. combined satellite and radar imagery to detect pockets of urban poverty in Rosario, Argentina [18]. Similar approaches to the management of information regarding slum settlements have been followed in a number of other settings, including Cape Town [19]. Finally, satellite imagery has been utilized to identify various settlement types in a variety of contexts, including informal homesteads in peri-urban America [20]. Whereas the studies that use satellite imagery in the wealthy country context (eg Ward and Peters 2007) have access to far richer and more easily linkable data, the mapping process used in the 2005 Bangladesh CMS provides a useful approach that could be applied to urban settings in other poor societies.

## Conclusion

The CMS has proven to be a popular tool for the practice of public health. Moreover, though it has become most well-known in the Bangladeshi public health community, the CMS should prove useful to organizations carrying out an array of programs in urban areas. It provides a rich source of information regarding the location and basic characteristics of slum communities in the major cities of Bangladesh, allowing programmers and planners to more precisely target their efforts toward areas of concentrated poverty and poor environmental conditions in a flexible fashion. The methodologies employed allowed a highly accurate set of maps to be produced relatively fast, providing timely information in a setting where circumstances can evolve quickly. It thus provides an important example of the valuable, real time information that can become available when more traditional field survey techniques are integrated with emerging geographic information tools.

## Competing interests

The authors declare that they have no competing interests.

## Authors' contributions

GA participated in the conceptual design and management of the census; analysis and interpretation of data; drafting of manuscript; critical revision and final manuscript approval. PML participated in the conceptual design and management of the census; analysis and interpretation of data; drafting of manuscript; critical revision and final manuscript approval. JBO participated in analysis and interpretation of data; drafting of manuscript; critical revision and final manuscript approval. NI participated in the conceptual design and management of the census; interpretation of data; manuscript revision and final manuscript approval. AQMM participated in the conceptual design and management of the census; interpretation of data; manuscript revision and final manuscript approval. NIN participated in the conceptual design and management of the census; interpretation of data; manuscript revision and final manuscript approval.

## Supplementary Material

Additional file 1 **Table S1 The Slums of the Six City Corporations: Tenure Security, Water, Electricity, and Sanitation**. Click here for file
