# Supplementary material for: The 2005 census and mapping of slums in Bangladesh: design, select results and application
Source: Int J Health Geogr. 2009 Jun 8;8:32. doi: 10.1186/1476-072X-8-32 (PMC2701942; doi:10.1186/1476-072X-8-32)
Supplement: Additional file 1 — Table S1 The Slums of the Six City Corporations: Tenure Security, Water, Electricity, and Sanitation. [file 1476-072X-8-32-S1.doc]

**Table S1 -The Slums of the Six City Corporations: Tenure Security, Water, Electricity and Sanitation**

| **City** | **Slums on public land*** | **Experience one or more evictions or currently under threat of eviction*** | **Drinking water from municipal tap*** | **Drinking water from tube well*** | **Drinking water source within slum*** | **With electricity*** | **With electricity**** | **Regular garbage collection*** | **Toilet linked to sewer/**  **septic tank **** | **Water sealed latrine**** |
| --- | --- | --- | --- | --- | --- | --- | --- | --- | --- | --- |
| Dhaka Metropolitan Area | 9.0 | 4.5 | 92.3 | 6.5 | 94.84 | 97.1 | 95.4 | 55.7 | 33.7 | 1.9 |
| Chittagong | 10.8 | 10.4 | 28.7 | 65.2 | 81.75 | 95.5 | 87.4 | 24.7 | 10.9 | 6.4 |
| Khulna | 12.5 | 17.5 | 2.1 | 97.9 | 58.85 | 91.5 | 72.5 | 19.6 | 1.0 | 9.1 |
| Rajshahi | 13.4 | 8.9 | 12.8 | 87.3 | 97.82 | 85.2 | 72.7 | 11.9 | 5.6 | 31.5 |
| Sylhet | 0.8 | 1.0 | 36.3 | 62.8 | 98.15 | 95.1 | 93.4 | 15.1 | 1.6 | 0.5 |
| Barisal | 11.1 | 5.1 | 15.6 | 84.4 | 78.06 | 98.6 | 95.8 | 7.4 | 0.0 | 0.4 |
| Total for Six Cities | 9.3 | 6.5 | 61.1 | 37.0 | 89.99 | 95.5 | 91.9 | 39.0 | 24.8 | 4.0 |
| * Percentage of slum communities.  **Percentage of households within slum communities. | | | | | | | | | | |

**Table S1 Continued**

| **City** | **Latrine not shared*** | **Latrines shared by 2-5 households*** | **Latrines shared by 6-10 households*** | **Latrines shared by 11-20 households*** | **Latrines shared by more than 20 households*** |
| --- | --- | --- | --- | --- | --- |
| Dhaka Metropolitan Area | 1.1 | 48.6 | 38.8 | 7.8 | 3.6 |
| Chittagong | 2.5 | 40.8 | 37.5 | 13.8 | 5.3 |
| Khulna | 1.5 | 41.9 | 42.9 | 11.1 | 2.5 |
| Rajshahi | 13.1 | 81.4 | 4.0 | 0.9 | 0.5 |
| Sylhet | 0.5 | 21.0 | 50.8 | 23.9 | 3.7 |
| Barisal | 8.0 | 70.9 | 17.1 | 2.6 | 1.4 |
| Total for Six Cities | 2.5 | 47.5 | 36.5 | 9.9 | 3.5 |
| * Percentage of slum communities.  **Percentage of households within slum communities. | | | | | |
